# Engineered *Saccharomyces cerevisiae* for High-Yield and Sustainable Production of α-Bisabolol via Combinatorial Genomic Integration and Pathway Amplification

**DOI:** 10.3390/jof12040251

**Published:** 2026-03-31

**Authors:** Zichen Wu, Baofeng Wan, Chun Li, Shen Zhou, Sishu Huang, Boyang Zhi, Congping Xu, Qin Cheng, Chuansong Zhan, Jie Luo

**Affiliations:** 1 Sanya Nanfan Research Institute of Hainan University, Sanya 572025, China; 2 Yazhouwan National Laboratory (YNL), Sanya 572025, China; 3 School of Life Science and Technology, Wuhan Polytechnic University, Wuhan 430023, China; 4 Sanya National Center of Technology Innovation for Saline-Alkali Tolerant Rice, Sanya 572024, China

**Keywords:** α-bisabolol, metabolic engineering, *Saccharomyces cerevisiae*, high-yield production, genomic integration

## Abstract

α-Bisabolol is naturally occurring in many plants and has great potential in health products and pharmaceuticals. However, the current extraction method from natural plants is unsustainable and cannot fulfil the increasing requirement. This study aimed to develop a sustainable strategy to enhance the biosynthesis of α-bisabolol by metabolic engineering. Integration of ERG20 (encoding farnesyl diphosphate synthase) and tHMG1 (encoding truncated 3-hydroxy-3-methylglutaryl-CoA reductase) genes with a constitutive strong promoter into the yeast genome elevated α-bisabolol production from 20.21 mg/L to 98.30 mg/L, representing a 4.86-fold increase. Further optimization of the mevalonate pathway and amplification of ERG20, tHMG1, and OsTPS1 copy numbers enhanced α-bisabolol synthesis to 423.01 mg/L, achieving a 20.93-fold improvement relative to the baseline. This work establishes a reference strategy for high-yield α-bisabolol biosynthesis in engineered yeast.

## 1. Introduction

Sesquiterpenoids constitute a significant class of natural products biosynthesized through the condensation of three isoprenoid precursor units: one dimethylallyl pyrophosphate (DMAPP) and two isopentenyl pyrophosphate (IPP) molecules [1,2]. Valued for their potent aromatic properties and notable anti-inflammatory activities, these compounds find broad applications across various industries [3,4]. Among them, α-bisabolol (C_15_H_26_O) is a monocyclic sesquiterpene alcohol initially isolated and extracted from *Matricaria recutita* [5,6]. Its pharmacological properties, including anti-inflammatory, antibacterial, skin-soothing, and moisturizing effects [7,8], render it a key ingredient in pharmaceutical and cosmetic formulations. Recent evidence suggesting analgesic functions indicates potential future clinical applications [9]. Furthermore, α-bisabolol exhibits low physiological toxicity [10,11], demonstrating considerable potential for widespread use as a health supplement.

α-Bisabolol occurs naturally in *Eremanthus erythropappus* and *Matricaria recutita* [3,12]. Traditional production relies on extraction and distillation processes from *E. erythropappus* leaves [3]. Nonetheless, the low yield of plant extraction and the low specificity of chemical catalysis limit the scope of α-bisabolol applications. In recent years, the advantages of utilizing microbial cell factories for α-bisabolol production have become increasingly attractive [13,14]. Despite this, industrial-scale production faces significant challenges due to low α-bisabolol titers.

A common biosynthetic strategy in engineered *Saccharomyces cerevisiae* employs inexpensive glucose as a carbon source to generate acetyl-CoA. This precursor enters the mevalonic acid (MVA) pathway to produce farnesyl pyrophosphate (FPP). Subsequently, FPP is converted to α-bisabolol by heterologously expressed α-bisabolol synthase, such as MrBBS from *M. recutita* [15,16].

Significant research efforts have recently focused on enhancing α-bisabolol production in microbial cell factories, yet numerous limitations and challenges persist. In *Escherichia coli*, overexpression of heterologous mevalonate pathway genes (mvaE, mvaS, mvaK1, mvaK2, mvaD, idi) and the farnesyl diphosphate synthase gene ispA improved the α-bisabolol titer to 9.1 g/L [17]. Employing the efficient α-bisabolol synthase CcBOS from *Cynara cardunculus* var. *scolymus* further increased the titer in *E. coli* to 23.4 g/L [18]. Compared to *E. coli*, fungi like yeast are less susceptible to phage infection. Optimization of the MVA pathway is crucial for enhancing α-bisabolol production in yeast. A recent study demonstrated that overexpression of tHMG1 (encoding truncated 3-hydroxy-3-methylglutaryl-CoA reductase), ERG10 (encoding acetoacetyl-CoA thiolase), and ACS1 (encoding acetyl-CoA synthetase) in *Saccharomyces cerevisiae* led to a 13-fold increase in α-bisabolol biosynthesis; however, the final production remained low at only 124 mg/L [15]. Consequently, developing novel α-bisabolol-producing yeast chassis based on *Saccharomyces cerevisiae* is expected to enable the sustainable and efficient production of α-bisabolol.

**Figure 1:**
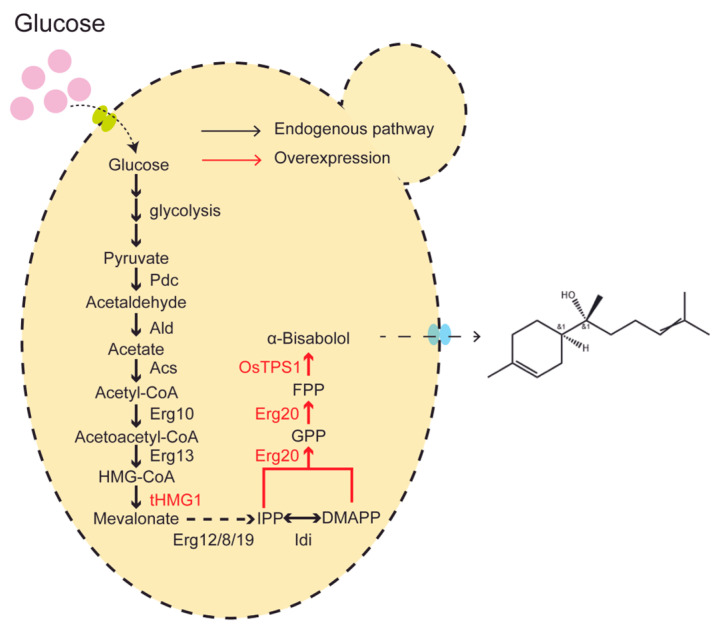
Metabolic engineering strategy for α-bisabolol production in *Saccharomyces cerevisiae*. The engineered pathway depicts key steps from central carbon metabolism (glucose) to α-bisabolol biosynthesis. Black arrows represent reactions catalyzed by the core endogenous mevalonate (MVA) pathway. Red arrows indicate heterologously introduced or enhanced enzymatic steps specific to bisabolol production. Abbreviations: Pdc, pyruvate decarboxylase; Ald, aldehyde dehydrogenase; Acs, acetyl-CoA synthetase; Erg10, acetyl-CoA C-acetyltransferase (thiolase); Erg13, hydroxymethylglutaryl-CoA synthase (HMG-CoA synthase); tHMG1, truncated hydroxymethylglutaryl-CoA reductase (HMG-CoA reductase); Erg12, mevalonate kinase; Erg8, phosphomevalonate kinase; Erg19, diphosphomevalonate decarboxylase; Idi, isopentenyl diphosphate isomerase; Erg20, farnesyl diphosphate synthase; OsTPS1, Oryza sativa α-bisabolol synthase (heterologous terpene synthase); IPP, isopentenyl diphosphate; DMAPP, dimethylallyl diphosphate; GPP, geranyl diphosphate; FPP, farnesyl diphosphate.

In this study, a series of engineered strains was constructed and validated to identify the bottleneck steps in α-bisabolol biosynthesis. Initially, integration of the heterologous α-bisabolol synthase gene OsTPS1 (from *Oryza sativa*) into the *Saccharomyces cerevisiae* genome increased α-bisabolol production from 14.8 mg/L (achieved using the episomal plasmid PESC- P_ADH1_-OsTPS1) to 20.2 mg/L. Subsequently, co-integration of tHMG1 and ERG20 (encoding FPP synthase) with OsTPS1 further enhanced the titer to 90.15 mg/L. By increasing the copy number of the OsTPS1, tHMG1, and ERG20 genes within the yeast genome, α-bisabolol production reached 423.01 mg/L (Figure 1). This sustainable production method holds significant potential for the industrial-scale manufacturing of α-bisabolol.

## 2. Materials and Methods

### 2.1. Chemicals, Strain, Plasmids, Primers and Culture Media

Yeast nitrogen base without amino acids and ammonium sulfate (YNB), Bacto peptone, Bacto yeast extract, LB medium, agar, lithium acetate and glucose were purchased from Solarbio, Beijing, China. Kanamycin, ampicillin, and amino acids (adenine, histidine, leucine, tryptophan, uracil) were purchased from Sigma-Aldrich, St. Louis, MO, USA. HPLC-grade methanol and acetonitrile were purchased from EMD Chemicals, Gibbstown, NJ, USA; HPLC-grade methanol was also obtained from Thermo Fisher Scientific, Waltham, MA, USA. The α-bisabolol standard was purchased from Sigma-Aldrich. Restriction enzymes, DNA polymerase, and dNTPs were purchased from Thermo Fisher Scientific; T4 DNA ligase was also used.

The assembly of promoter module P1 (P_TDH3_-P_ADH1_) exemplifies promoter module construction. Promoters P_TDH3_ and P_ADH1_ were fused in a divergent orientation using overlap PCR. The amplified fragment was subsequently inserted into the pMD19T-Amp TA cloning vector between the M13F and M13R sites. Synthetic terminator cassettes (e.g., L1-T_PDC1_-P_ADH1_-L2 for T1-T_FBA1_-P_PGK1_) were commercially synthesized (GENEWIZ, Beijing, China). These cassettes were then cloned into either the pMD19T-Amp or pUC57-kan plasmid backbone. Homologous recombination arms, designated Site1 (~500 bp upstream of the target genomic integration locus) and Site2 (~500 bp downstream), were employed. A DNA fragment containing Site1 fused to the URA3 selectable marker and L1 linker was generated by overlap PCR. This Site1-URA3-L1 cassette was cloned into the pUC57-Kan vector for storage. Site2 cassettes were constructed following an analogous procedure. The strains and plasmids are listed in Supplementary Table S1, the Primers are listed in Supplementary Table S2 and the sequences in this study are listed in Supplementary Table S3.

*Saccharomyces cerevisiae* BY4741 (genotype: MATa his3Δ1 leu2Δ0 met15Δ0 ura3Δ0) served as the parental strain for all engineered yeast strains. pMD-19T plasmids served as the vectors for assembly [19]. *E. coli* strains were cultured in LB medium supplemented with appropriate antibiotics. Yeast strains were cultured either in synthetic dropout (SD) medium lacking leucine, uracil, tryptophan, and histidine, or in YPD medium. SD medium was used during the shake-flask fermentation stage.

Three single colonies were first cultured in 15 mL centrifuge tubes containing 4 mL medium at 30 °C and 220 rpm until reaching an OD_600_ ≈ 1.0. This seed culture was then used to inoculate 150 mL Erlenmeyer flasks containing 50 mL medium, achieving an initial OD_600_ of 0.05. The main cultures were incubated for 5 days at 30 °C and 220 rpm. The resulting products were analyzed by GC-MS after extraction.

### 2.2. Yeast Transformation and Genomic Integration

For genomic integration, the selectable marker module along with the integration homology arm module was introduced into *Saccharomyces cerevisiae* strain BY4741 via electroporation. Positive transformants were subsequently cultured in 50 mL of YPD medium within 150 mL Erlenmeyer flasks at 30 °C with shaking at 220 rpm. Competent cells were prepared as follows: Three single colonies were inoculated into 4 mL of liquid YPD medium and incubated until reaching an OD_600_ of 0.6–0.8. A 2 mL aliquot of the culture was centrifuged at 10,000× *g* for 1 min to harvest cells. The resulting cell pellet was washed twice with 1 mL of ice-cold sterile water and subsequently resuspended in 1 mL of transformation buffer (10 mM LiAc, 10 mM DTT, 0.6 M sorbitol, 10 mM Tris–HCl, pH 7.5). Following a 20 min incubation at 25 °C, the treated cells were pelleted by centrifugation, washed twice with 1 mL of ice-cold 1 M sorbitol solution, and finally resuspended in 100 μL of 1 M sorbitol solution.

A 100 μL aliquot of competent cells was mixed with 200–500 ng of DNA. Electroporation was performed using a Bio-Rad Gene Pulser (Hercules, CA, USA) under the following conditions: 2.7 kV, 25 μF, and 200 Ω. Immediately after electroporation, 1 mL of 1 M sorbitol solution was added to the cell suspension. The mixture was incubated at 30 °C for 2 h and then plated onto selective medium. Transformants were allowed to grow for 3 days.

### 2.3. GC-MS Analysis and PCR Verification of Pathway Assembly

α-bisabolol was extracted using n-hexane as the solvent. The pure α-bisabolol (CAS: 515-69-5) was purchased from TCI (tcichemicals, https://www.tcichemicals.com/CN/zh/p/B2119, accessed on 9 June 2023). Separation was performed on a Shimadzu SH-Rtx-5MS capillary column (Shimadzu, Tokyo, Japan) (30 m length × 0.25 mm internal diameter, 0.25 μm film thickness). The oven temperature was programmed starting at 80 °C, with a ramp rate of 15 °C min^−1^. Injection was performed in spitless mode. Mass spectrometry was conducted using an electron ionization (EI) source operated at 70 eV, scanning the mass range from *m*/*z* 50 to 500 Da. Helium was used as the carrier gas.

Single colonies were inoculated into 4 mL of SC-Ura liquid medium and cultured overnight at 30 °C with shaking at 250 rpm. Cells were harvested by centrifugation, and genomic DNA was extracted using a Yeast Genomic DNA Extraction Kit (CWBiotech, Taizhou, China). PCR amplification was performed using 2 μL of total DNA as template and 2× Taq Master Mix (CWBiotech, China).

### 2.4. Stability Analysis of the Engineered Saccharomyces cerevisiae

To evaluate the stability of the constructed strain, bis03 was subjected to subculture. Three single colonies were individually inoculated into test tubes containing 50 mL of YPD medium, followed by continuous subculturing for 20 generations. The cultures of the 20th generations were separately used as seed cultures for shake-flask cultivation. The genetic stability of the engineered yeast strain bis03 was preliminarily assessed by PCR verification of the integration loci of the synthetic pathway and determination of α-bisabolol yield.

## 3. Results

### 3.1. Stable Genomic Integration of OsTPS1 Enhances α-Bisabolol Production

**Figure 2:**
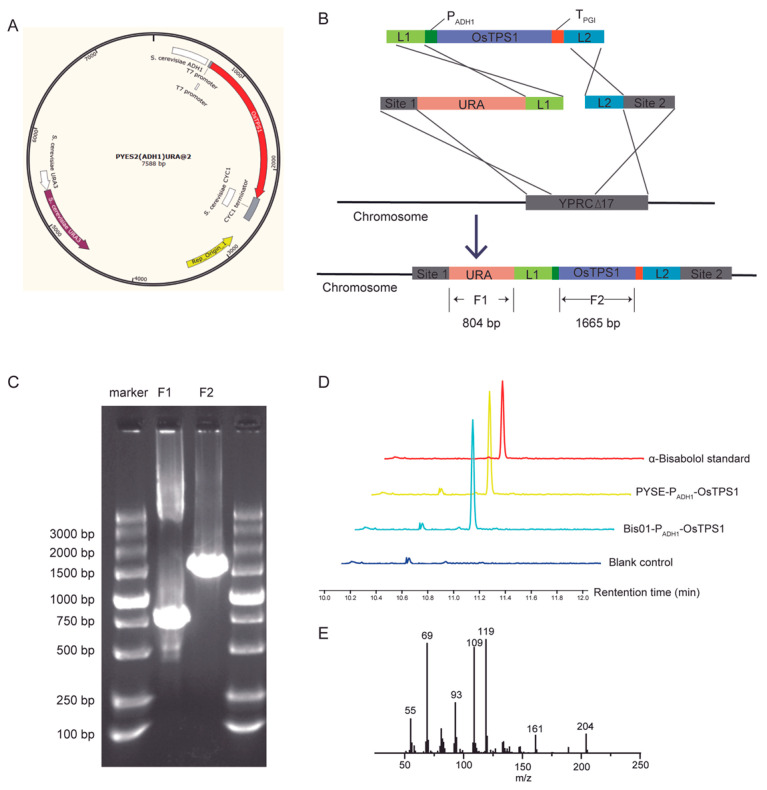
Assembly, integration, and functional validation of the α-bisabolol biosynthetic pathway. (**A**) Vector map of PESC- P_ADH1_-OsTPS1. (**B**) Schematic of the integrated α-bisabolol biosynthetic pathway at the YPRCΔ17 locus. (**C**) Agarose gel electrophoresis of PCR products confirming pathway assembly and integration at the YPRCΔ17 locus. Lanes: M, DNA ladder; 1, F1 product; 2, F2 product. (**D**) GC-MS chromatogram demonstrating α-bisabolol production in the engineered strain. (**E**) Mass spectrum of the α-bisabolol (**D**).

To evaluate the relative effectiveness of episomal plasmids versus genomic integration in yeast, we constructed the PESC-P_ADH1_-OsTPS1 plasmid and the L1-P_ADH1_-OsTPS1-L2 plasmid (where P_ADH1_ denotes the promoter of the alcohol dehydrogenase gene) using seamless cloning Figure 2A. Following successful plasmid construction, the PESC-P_ADH1_-OsTPS1 plasmid was transformed into the BY4741 yeast strain, obtaining a transformant harboring episomal plasmid. Additionally, three DNA fragments amplified from the corresponding vector—L1-P_ADH1_-OsTPS1-L2, L1- URA3-Site1, and L2-Site2—were transformed into the BY4741 strain (Figure 2B).

To confirm the successful genomic integration of the relevant fragments, genomic DNA was extracted from selected yeast colonies and subjected to PCR verification. As shown in Figure 2C, PCR amplification using primer pairs yielded fragments of the expected sizes for the integrated URA3 gene (F1) and OsTPS1 gene (F2), respectively, in the engineered yeast strain Bis01.

Positive transformants were then cultured in 50 mL of YPD medium within 150 mL Erlenmeyer flasks at 30 °C with shaking. Compared to the strain harboring the episomal plasmid PESC-P_ADH1_-OsTPS1, the engineered yeast strain with genomically integrated OsTPS1 exhibited an increase in α-bisabolol production from 14.8 mg/L to 20.2 mg/L (Figure 2D; Figure S1 in Supporting Information).

To verify that the compound synthesized by the introduction of OsTPS1 was indeed α-bisabolol, we performed metabolite extraction from the fermentation broth of the OsTPS1-expressing yeast strain followed by analysis using Gas Chromatography-Mass Spectroscopy (GC-MS). The GC-MS peak corresponding to the extracted metabolite aligned precisely with that of the authentic α-bisabolol standard (Figure 2E). This confirmed the successful synthesis of α-bisabolol in yeast.

### 3.2. Combinatorial Integration of MVA Pathway Genes Synergistically Boosts Production

To further enhance α-bisabolol production in yeast, we hypothesized that increasing the synthesis of its precursor compounds could be beneficial. As the precursor farnesyl pyrophosphate (FPP) is primarily derived from the mevalonate (MVA) pathway, and hydroxymethylglutaryl-CoA reductase (HMGR) is the rate-limiting enzyme of this pathway, we proposed that simultaneous genomic integration of tHMG1 (encoding a truncated, deregulated form of HMGR), OsTPS1 (encoding α-bisabolol synthase), and ERG20 (encoding FPP synthase, the direct precursor synthesizing enzyme) could achieve this goal.

**Figure 3:**
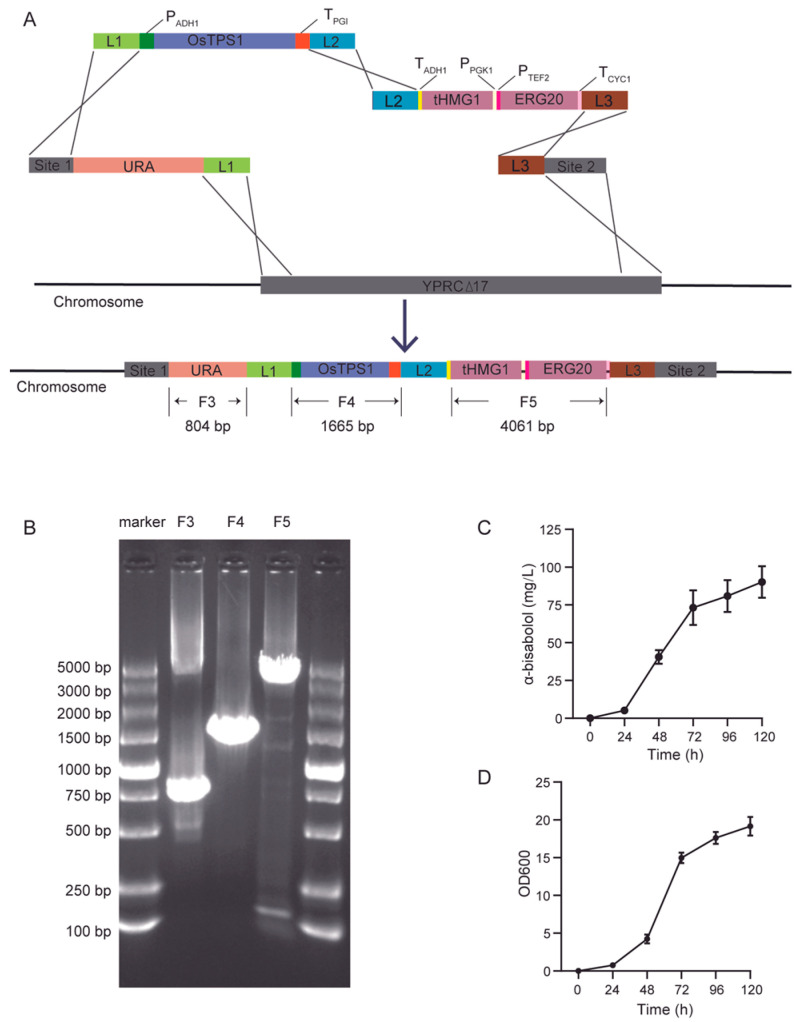
Assembly, integration, and functional validation of the four-gene α-bisabolol biosynthetic pathway. (**A**) Schematic of the integrated four-gene pathway at the YORWΔ17 locus. (**B**) Agarose gel electrophoresis of PCR verification for pathway assembly and integration. F3 to F5 indicate four PCRs designed to confirm correct assembly and integration. (**C**) α-Bisabolol production by engineered strain bis02-OsTPS1-tHMG1-ERG20. Data represent mean ± SD of triplicate cultivations in 150-mL Erlenmeyer flasks. (**D**) Growth profile (OD_600_) of strain bis02-OsTPS1-tHMG1-ERG20 under conditions in (**C**).

Accordingly, we constructed the plasmids L1-P_ADH1_-OsTPS1-L2 and L2-tHMG1-P_PGK1_-P_TEF2_-ERG20-L3 using seamless cloning. Following successful plasmid construction, the corresponding DNA fragments for yeast transformation were amplified via PCR. Four DNA fragments containing the target genes—L1- URA3-Site1, L1-P_ADH1_-OsTPS1-L2, L2-tHMG1-P_PGK1_-P_TEF2_-ERG20-L3, and L3-Site2—were co-transformed into the BY4741 strain. (Figure 3A).

To confirm successful genomic integration of the relevant fragments, genomic DNA was extracted from selected yeast colonies and subjected to PCR verification. As shown in Figure 3B, PCR amplification of the engineered strain Bis02 using primer pairs yielded DNA fragments of the expected sizes corresponding to the integrated URA3 gene (F3), OsTPS1 gene (F4), and the tHMG1-P_PGK1_-P_TEF2_-ERG20 cassette (F5), respectively.

Positive transformants were subsequently cultured in 50 mL of YPD medium within 150 mL erlenmeyer flasks at 30 °C with shaking at 220 rpm. α-bisabolol production in YPD medium by positive transformant was evaluated. Compared to the Bis01 yeast strain, the engineered Bis02 strain exhibited a significant increase in α-bisabolol production, rising from 20.2 mg/L to 90.15 mg/L, representing a 4.46-fold enhancement (Figure 3C,D).

### 3.3. Multi-Copy Amplification of Key Genes Drives Exceptional Titer Enhancement

**Figure 4:**
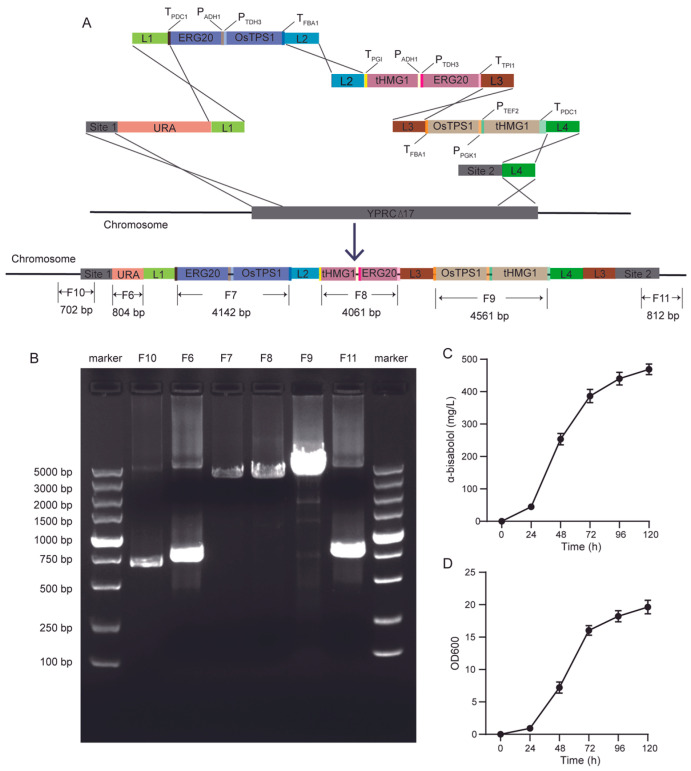
Assembly, integration, and functional validation of the seven-gene α-bisabolol biosynthetic pathway. (**A**) Schematic of the integrated seven-gene pathway at the YORWΔ17 locus. (**B**) Agarose gel electrophoresis of PCR verification for pathway assembly and integration. F6 to F11 indicate four PCRs designed to confirm correct assembly and integration. (**C**) α-Bisabolol production by engineered strain bis03-ERG20-OsTPS1-tHMG1-ERG20-OsTPS1-tHMG1. Data represent mean ± SD of triplicate cultivations in 150-mL Erlenmeyer flasks. (**D**) Growth profile (OD_600_) of strain bis03-ERG20-OsTPS1-tHMG1-ERG20-OsTPS1-tHMG1 under conditions in (**C**).

To further enhance α-bisabolol production in yeast, we hypothesized that increasing the copy number of the α-bisabolol synthase gene could be beneficial. Using seamless cloning, we successfully constructed the plasmids L1-ERG20-P_ADH1_-P_TDH3_-OsTPS1-L2, L2-tHMG1-P_ADH1_-P_TDH3_-ERG20-L3, and L3-OsTPS1-P_PGK1_-P_TEF2_-tHMG1-L4. Following successful plasmid construction, the corresponding DNA fragments for yeast transformation were amplified via PCR. Five DNA fragments containing the target genes—L1- URA3-Site1, L1-ERG20-P_ADH1_-P_TDH3_-OsTPS1-L2, L2-tHMG1-P_ADH1_-P_TDH3_-ERG20-L3, and L3-OsTPS1-P_PGK1_-P_TEF2_-tHMG1-L4, and L4-Site2—were co-transformed into the BY4741 strain, and Bis 03 transformant was obtained (Figure 4A).

To confirm successful genomic integration of the relevant fragments, genomic DNA was extracted from selected yeast colonies and subjected to PCR verification. As shown in Figure 4B, PCR amplification of the engineered strain Bis03 using primer pairs yielded DNA fragments of the expected sizes corresponding to the integrated URA3 gene (F6), the ERG20-P_ADH1_-P_TDH3_-OsTPS1 cassette (F7), the tHMG1-P_ADH1_-P_TDH3_-ERG20 cassette (F8), and the OsTPS1-P_PGK1_-P_TEF2_-tHMG1 cassette (F9), partly left DNA sequence flanking YORW site and partly sequence of Site 1 (F10), partly sequence of Site 2 and partly right DNA sequence flanking (F11) respectively.

Compared with the Bis02 yeast strain, the engineered Bis03 strain exhibited a significant increase in α-bisabolol production, rising from 90.15 mg/L to 423.01 mg/L, which represented a 4.69-fold enhancement. After 20 generations of subculture, the integrated genes at the target loci could still be detected by PCR in the engineered strain, and the α-bisabolol production remained nearly unchanged (Figure 4B–D). These results demonstrated that the bis03 strain was genetically stable during subsequent subcultures.

## 4. Discussion

This study demonstrates a systematic and highly effective approach for engineering *Saccharomyces cerevisiae* to achieve high-level production of the valuable sesquiterpene alcohol α-bisabolol. By progressively integrating key biosynthetic genes into the yeast genome and optimizing their copy number, we achieved a remarkable final titer of 423.01 mg/L in shake-flask culture, representing a significant advancement in microbial α-bisabolol production.

Our initial step involved the stable chromosomal integration of the heterologous α-bisabolol synthase gene (OsTPS1) [3], replacing episomal plasmid-based expression. This resulted in a 36.5% increase in titer (from 14.8 mg/L to 20.2 mg/L, strain Bis01), confirming the functional expression of OsTPS1 and highlighting the inherent advantages of genomic integration, such as enhanced genetic stability and elimination of plasmid maintenance burdens, for long-term fermentation processes [20]. This foundational strategy aligns with the broader goal of developing robust and economically viable microbial cell factories.

Recognizing the critical role of precursor supply, we next engineered the native mevalonate (MVA) pathway to enhance flux towards farnesyl pyrophosphate (FPP), the direct precursor of α-bisabolol. The simultaneous genomic integration of tHMG1 (encoding a truncated, deregulated hydroxymethylglutaryl-CoA reductase, HMGR) and ERG20 (encoding FPP synthase), alongside OsTPS1 (strain Bis02), yielded a dramatic 4.46-fold increase in production (90.15 mg/L). This substantial improvement underscores the rate-limiting nature of HMGR within the MVA pathway [21] and validates the strategy of deregulating HMGR (tHMG1) to overcome this bottleneck [22]. Furthermore, the co-integration of ERG20 ensures efficient conversion of the pathway intermediates IPP and DMAPP into the required FPP substrate. This combinatorial enhancement of precursor supply and terpene synthase expression is consistent with successful strategies employed for other sesquiterpenes and directly addresses the challenge of insufficient FPP availability. The success of this step also implicitly supports the notion that coordinated upregulation of multiple pathway genes is often more effective than modulating single non-limiting genes [21].

The most significant leap in titer, however, was achieved through multi-copy genomic integration of the key biosynthetic cassettes, strain Bis03. Integrating additional copies of tHMG1, ERG20, and crucially, OsTPS1, boosted production to 423.01 mg/L, a further 4.69-fold increase over Bis02. This result strongly emphasizes the critical impact of gene dosage on metabolic flux. Increasing the copy number of OsTPS1 directly enhances the capacity to convert the augmented FPP pool into the target product. Similarly, higher copy numbers of tHMG1 and ERG20 ensure sustained high flux through the MVA pathway towards FPP synthesis. This strategy proved remarkably effective, overcoming potential limitations seen with weaker promoters or single-copy integrations. The careful design of the integration cassettes, utilizing strong constitutive promoters (P_TDH3_, P_TEF2_, P_PGK1_) known for reliable high expression in yeast, further contributed to maximizing enzyme levels. This finding resonates with studies highlighting promoter strength and copy number as key determinants of terpenoid titers in engineered microbes [20,23]. The near-29-fold overall increase from the initial episomal expression strain to Bis03 vividly demonstrates the cumulative power of genomic integration, pathway engineering, and copy number amplification.

While achieving high intracellular production is essential, efficient transport of α-bisabolol out of the cell is crucial to alleviate potential product toxicity or feedback inhibition and to maximize titers in the fermentation broth [24,25]. Our engineered strains achieved significant production without explicit transporter engineering in this specific study. However, the background text highlights the proven efficacy of overexpressing endogenous ABC transporters like PDR15 in enhancing the efflux of α-bisabolol and other lipophilic compounds [26,27]. Future work will prioritize exploring transporter engineering (e.g., PDR15 overexpression) as a key strategy to further improve yields, particularly as titers increase towards industrial levels, where intracellular accumulation might become inhibitory [28,29]. Implementing two-phase fermentation systems could also synergistically enhance product secretion and recovery [29].

Despite the impressive titer achieved in shake flasks (423.01 mg/L), several avenues remain for further optimization to reach the gram-per-liter scale demonstrated for other terpenes in bioreactors. Firstly, precursor availability upstream of FPP, particularly acetyl-CoA, can become limiting under high flux conditions. Systematic engineering of the acetyl-CoA synthesis pathway, potentially involving strategies to redirect carbon flux away from ethanol production, presents a clear opportunity [21,30]. Secondly, the intensified MVA pathway flux driven by high tHMG1 and ERG20 expression imposes a significant demand for the cofactor NADPH. Enhancing NADPH regeneration capacity through metabolic engineering could alleviate this potential limitation and further boost pathway efficiency [21,31]. Thirdly, fine-tuning the expression balance between ERG20 and OsTPS1, potentially exploring fusion proteins as successfully applied for geraniol [32] and valencene [22], might optimize substrate channeling from FPP to α-bisabolol, minimizing potential FPP diversion or loss. Finally, as mentioned, transporter engineering and advanced fermentation strategies (fed-batch, two-phase) will be essential for scaling up production and achieving economically viable titers [26,28]. Replacing the URA3 selection marker with a non-auxotrophic alternative would also enhance the industrial applicability of the strain.

## 5. Conclusions

In conclusion, we have successfully engineered *Saccharomyces cerevisiae* for high-level de novo production of α-bisabolol through a progressive strategy of stable genomic integration and copy number amplification of the key genes OsTPS1, tHMG1, and ERG20. The stepwise engineering approach, culminating in a titer of 423.01 mg/L in shake flasks, demonstrates the profound impact of enhancing precursor supply (via tHMG1 and ERG20) and catalytic capacity (via OsTPS1), particularly through increased gene dosage. This work establishes a robust foundation for α-bisabolol production in yeast. The strategies employed—genomic integration, MVA pathway enhancement, and copy number optimization—are broadly applicable to the microbial production of other valuable terpenoids. Future efforts focusing on acetyl-CoA supply, NADPH regeneration, transporter engineering, and advanced fermentation processes promise to further elevate titers towards industrial feasibility.

## Data Availability

The original contributions presented in this study are included in the article/Supplementary Material. Further inquiries can be directed to the corresponding authors.

## Supplementary Materials

The following supporting information can be downloaded at: https://www.mdpi.com/article/10.3390/jof12040251/s1, Figure S1: α-Bisabolol biosynthesis in PESC-P_ADH1_-OsTPS1-transformed strain and engineered strain Bis01-P_ADH1_-OsTPS1. Data represent mean ± SD of triplicate cultivations in 150-mL Erlenmeyer flasks. * *p* < 0.05 by unpaired two-tailed Student’s *t*-tests. Table S1: Strain and plasmids used in this study; Table S2: Primers used in this study; Table S3: List of sequences in this study.
