# Supplementary material for: Engineered Saccharomyces cerevisiae for High-Yield and Sustainable Production of α-Bisabolol via Combinatorial Genomic Integration and Pathway Amplification
Source: J Fungi (Basel). 2026 Mar 31;12(4):251. doi: 10.3390/jof12040251 (PMC13118236; doi:10.3390/jof12040251)
Supplement: Supplementary file 1 [file jof-12-00251-s001.zip › jof-4215827-supplementary.pdf]

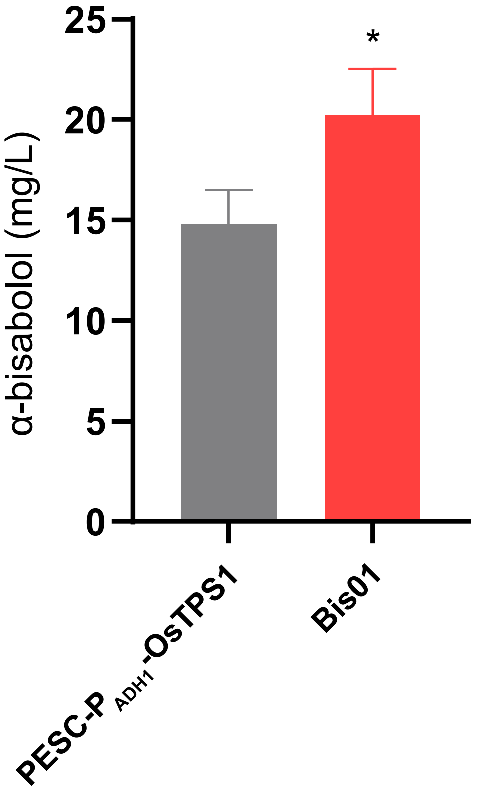

Figure S1. α-Bisabolol biosynthesis in PESC-P_{ADH1}-OsTPS1-transformed strain and engineered strain Bis01-P_{ADH1}-OsTPS1. Data represent mean ± SD of triplicate cultivations in 150-mL Erlenmeyer flasks. *$P < 0.05$ by unpaired two-tailed Student's *t*-tests.

Table S1. Strain and plasmids used in this study

**Strains**	**Host strain**	**Description**	**Reference of source**
BY4741	BY4741	MATa his3Δ1 leu2 met15Δ ura3-52	Lab collection
Bis01	BY4741	YORW△17::URA3/P_{ADH1}-OsTPS1-T_{ADH1}	This study
Bis02	BY4741	YORW △ 17::URA3/PADH1-OsTPS1-TADH1/PADH1-tHMG1-TPGI/PTDH3-ERG20-TTPI1	This study
Bis03	BY4741	YORW △ 17::URA3/PADH1-ERG20-	This study

		TPDC1/PTDH3-OsTPS1-TFBA1/PADH1-tHMG1-TPGI/PTDH3-ERG20-TTPI1/PTEF2-OsTPS1-TCYC1/PPGK1-tHMG1-TADH1	
**Plasmids**			This study
T1-(PADH1-TPGI)		pUC57, KanR, Homologous arm L1 and L2	This study
T1-(TPGH1-PTDH3-PADH1-TPG1)		pMD-19T, AmpR, PADH1, TPDC1, Homologous arm L1 and L2	This study
T2-(PPGK1-TADH1-PTEF2-TCYC1)		pUC57, KanR, Homologous arm L2 and L3	This study
T3-(PTDH3-TFBA1-PADH1-TPDC1)		pUC57, KanR, Homologous arm L3 and L4	This study
T1-OsTPS1		T1 vector, PADH1-OsTPS1-TADH1	This study
T1-ERG20-OsTPS1		T1 vector, PADH1-ERG20-TPDC1/PTDH3-OsTPS1-TFBA1	This study
T2-tHMG1-ERG20		T2 vector, PADH1-tHMG1-TPGI/PTDH3-ERG20-TTPI1	This study
T3-OsTPS1-		T3 vector, PTEF2-OsTPS1-TCYC1/PPGK1-	This study

tHMG1		tHMG1-TADH1	

Table S2. Primers used in this study

Primer Name	Primer Sequence
F-ADH1-OsTPS1	tcaactatctcatatacaATGCCTGCTGATCATATGGAA
R-PGI-OsTPS1	ATATTTAAGAGCGATTTGTTTCACACTTGTATTGGAACCACATAGATC
F-TDH3-ERG20	CACACATAAACAAACAAAATGGCTTCAGAAAAAGAAATTAGGAGA
F-TPI1-ERG20	TTATATAATTATATTAATCCTATTTGCTTCTCTTGTAAACTTTGTTC
F-ADH1-OsTPS1	aatcaactatctcatatacaATGCCTGCTGATCATATGGAAAAGA
R-PGI-OsTPS1	TATATTTAAGAGCGATTTGTTTCACACTTGTATTGGAACCACATAGATC
F-PGK1-tHMG1	TACAACAAATATAAAACAATGGcTGCAGACCAATTGGTGAAAAC
R-ADH1-tHMG1	CTTATTTTTTTATAACTTTAGGATTTAATGCAGGTGACGGACCCATC
F-TEF2-	TTAACTAAACACTAGTACCATGGCTTCAGAAAAAGAAATTAGGAGA

ERG20	G
R-CYC1-ERG20	gacataactaattacatgaCTATTTGCTTCTCTTGTAAACTTTGTTCAAGAA
F-PGK1-OsTPS1	ACAACAAATATAAAACAATGCCTGCTGATCATATGGAAAAGAC
R-FBA1-OsTPS1	ATTAATTTGAATTAACaagcttTCACACTTGTATTGGAACCACATAGATCGATGA
F-TEF2-tHMG1	TAATTAACTAAACACTAGTACCATGGcTGCAGACCAATTGGTGAAAACTGAAGT
R-PDC1-tHMG1	GAGATTAAATCGCgaattcTTAGGATTTAATGCAGGTGACGGACCCATCT

Table S3. List of sequences in this study

P_{ADH1}	acatgtaggtggcggaggggagatatacaatagaacagataccagacaagacataatgggctaaacaagactacaccaattacactgcctcattgatggtggtacataacgaactaatactgtagccctagacttgatagccatcatcatatcgaagtttcactacccttttccatttgccatctattgaagtaataataggcgcatgcaacttcttttcttttttttcttttctctctcccccgttgttgtctcaccatatccgcaatgacaaaaaaatgatggaagacactaaaggaaaaaattaacgacaaagacagcaccaacagatgtcgttgttccagagctgatgaggggtatctcgaagcacacgaaactttttccttccttcattcacgcacactactctctaatgagcaacggtatacggccttccttccagttacttgaatttgaaataaaaaaaagtttgctgtcttgctatcaagtataaatagacctgcaattattaatcttttgtttcctcgtcattgttctcgttccctttcttccttgtttctttttctgcacaatatttcaagctataccaagcatacaatcaactatctcatataca

OsTPS1	ATGCCTGCTGATCATATGGAAAAGACCGTGCCAACAGTTGTTCCTGCTCGAC<br>CTCCCAACAGCGAGGGGGAGCGGAAGTCAACCAACTTCCATCCTAGCTTGT<br>GGGGCGATTTCTTCCTCAACTATGAGCCGCCAACTGCGCCACAGCAAGCAT<br>GCATGAAAGGAAGGGCTGAAGTGTTGAGAGAAGAAGTTAGAACATTATTAA<br>AGGGTTTAAAGGAAGTACAAAAAATATTGGACATTACAATGGCAATACAAC<br>GGTTGGGACTAGACATGTACTATGTGAACGAGATTAATGAGCTGTTGCACTT<br>TGTTTACAGCTCTGATTACAATGACAAAGATTTGAATTTAGTTTCACTTCGAT<br>TTTATCTTCTGCGAAAGAATGGCTACAATATGTCATCCGGTATTTTTCTCAGTT<br>TTAAAGACAATGAAGGGAATTTCATTGTTGATGACACAAGAACTCTGTTAAA<br>CTTATATAATGCAGCAAACCTTAGGGTTTATGGAGAGAAGGTGCTTGATGAG<br>GCTGCAACTTTCACTATAAGTCGCCTTGAGGGTGTGTTGGAATCATCAGACT<br>CAATATTATCAACGGAAGTATCCTTTGCCCTTGAAGCACCAATATTCCGAAG<br>GGCACGAATTGTAGAAATGAGAAACTACATTCCTATTTATGAAATAGAGGCT<br>ACAAGAAATGAAACCATATTGGAGTTTGCGAAACTGAATTTCAACCTTCTGC<br>AACTTCTTTACTGTGAGGAATTAAATAAGATCACATTGTGGTGGAAGGAGCT<br>TAAAGTCAAATCAAACTTGAGTTTTTCTAGAGATAGGATAGTAGAAATGTAT<br>TTTTGGATGAATGGAGCACTATATGAACCTCATTACTCTCaCTCCAGAATTATA<br>CTTACAAGGGTGACAGCATTTATGACAATAATAGATGATATATTTGACACATA<br>TGGTACAACAGAAGAGAGTATG<br>CTACTTGCTGAAGCAATTAATAGGTGGGATGAAAGTGCAATAGGACTGCTCC<br>CAGAGTATATTAGGGGGTTCTATGCATACTTGTTGAAGACATTTGACTCATTC<br>GAGGAAGAGCTTGCACCTGAGAAGAGATACCGTGTATTTTATCTAAAAGAA

	ATGCTAAAACAGTTAGTTCAGGCCTACACCAAGGAGTTAAAATGGCGTGAC<br>GAGGATTATACGCCAAAAACGTTGGAGGAACATTTTGAGGTTTCAATGAGA<br>AGTAGTGGTGGTTTTACATTAGCAGCTGCTTCATTTGTTGGAATGGATGACAT<br>AGCAACCAAAGACATATTTGAATGGATTTTGAGTTATCCATCCCTTTTCAAGA<br>CTTTTGATATATTTGTGCGACTCTCCAATGATATTGTATCAAATAAGCGTGAGC<br>AAACTGGGGACCACTATGCCTCTACAATTCAATGCTACATGAAGGAGCATGG<br>GACAACAATTCATGAGACCTACCAAAGGTTAAGAGAACTCATCGAAGACTC<br>ATGGAAGGATATGGTAGAACATTGCACAAACCCGATTGATGACCAACCATT<br>GATCGTGCCCCAGACTGTAGTGAACTTCGCAAGGACAGTGACAACCATGTA<br>TACCCACGGTGATGCATTCACTTCTTCACACACAATCAAGGAAATGATATCA<br>TCGATCTATGTGGTTCCAATACAAGTGTGA
T_{PGI}	AACAAATCGCTCTTAAATATATACCTAAAGAACATTAAAGCTATATTATAAGC<br>AAAGATACGTAAATTTTGCTTATATTATTATACACATATCATATTTCTATATTTTT<br>AAGATTTGGTTATATAATGTACGTAATGCAAAGGAAATAAATTTTATACATTAT<br>TGAACAGCGTCCAAGTAACTACATTATGTGCACTAATAGTTTAGCGTCGTGA<br>AGACTTTATTGTGTCGCGAAAAGTAAAAATTTTAAAAATTAGAGCACCTTGA<br>ACTTGCGAAAAAGGTTCTCATCAACTGTTTAAAAGGAGGATATCAGGTCCTA<br>TTTCTGACAAACAATATACAAATTTAGTTTCAAAGATGAATCAGTGCGCGAA<br>GGACATAACTCATGAAGCCTCCAGTATACC
L1	CGTCTCCCCCGGTCCGTTTGTTCTATACTTCTCTCTGCTATACCTACAAGCAA<br>GGTAATCGGAAGTAGTATTACGCAGGAATATCCCGCGCGAAGCTACAATTTT<br>TGGACTCCAACGTCAAAGCAGGGGAGTCAGAAGTCCCCTCTAAAATTGCCT

L2	GACAAAGCGCCAAGGAACTGTAATATATAGCTACGCCCTATCTGGACGATTG<br>GGCGACTTTTACGTACGGTTGCTCAATTCCTACGCAACTTAATATATTTTGCA<br>ACGGTTAAATCGGCTTGAAGCTCGGGCTATCCAACTCGCGGACTA
T_{TPI1}	CTATATAACAGTTGAAATTTGGATAAGAACATCTTCTCAACGCGAAAATGAC<br>GCCTCCAGTGAAAAAACATAAACTTTCAATGCAGTCTTCGGTACACTTATGA<br>GTAACCCATATAGAGATCGTACACATTTTACAAGGATTTAGAGACAAAGTAA<br>TATTCTTCGTTGATATAGAGGTGTTCAATTGTTAAATGCTTTTCTTCTTTTTATT<br>AGAAAAAGCGCCTTGCTTTTTGTTGTATCACTTGTAAATCTACCGTCCCTTAC<br>AAGAACATTCACGAAATTTAAGTGGCTCAGAATGAAAAAGAAACAATATAA<br>AAAAG<br>CTTTCCGTAGTCATCAATTTATTTTACATAACACTAGATATAAAGAAAAGAAG<br>ATAATATTTTTATATAATTATATTAATC
ERG20	ATGGCTTCAGAAAAAGAAATTAGGAGAGAGAGATTCTTGAACGTTTTCCCT<br>AAATTAGTAGAGGAATTGAACGCATCGCTTTTGGCTTACGGTATGCCTAAGG<br>AAGCATGTGACTGGTATGCCCACTCATTGAACTACAACACTCCAGGCGGTA<br>AGCTAAATAGAGGTTTGTCCGTTGTGGACACGTATGCTATTCTCTCCAACAA<br>GACCGTTGAACAATTGGGGCAAGAAGAATACGAAAAGGTTGCCATTCTAGG<br>TTGGTGCATTGAGTTGTTGCAGGCTTACTTCTTGGTCGCCGATGATATGATGG<br>ACAAGTCCATTACCAGAAGAGGCCAACCATGTTGGTACAAGGTTCCTGAAG<br>TTGGGGAAATTGCCATCAATGACGCATTCATGTTAGAGGCTGCTATCTACAA<br>GCTTTTGAAATCTCACTTCAGAAACGAAAAATACTACATAGATATCACCGAA<br>TTGTTCCATGAGGTCACCTTCCAAACCGAATTGGGCCAATTGATGGACTTAA

	TCACTGCACCTGAAGACAAAGTCGACTTGAGTAAGTTCTCCCTAAAGAAGC<br>ACTCCTTCATAGTTACTTTCAAGACTGCTTACTATTCTTTCTACTTGCCTGTCG<br>CATTGGCCATGTACGTTGCCGGTATCACGGATGAAAAGGATTTGAAACAAG<br>CCAGAGATGTCTTGATTCCATTGGGTGAATACTTCCAAATTCAAGATGACTA<br>CTTAGACTGCTTCGGTACCCCAGAACAGATCGGTAAGATCGGTACAGATATC<br>CAAGATAACAAATGTTCTTGGGTAATCAACAAGGCATTGGAACTTGCTTCCG<br>CAGAACAAAGAAAGACTTTAGACGAAAATTACGGTAAGAAGGACTCAGTC<br>GCAGAAGCCAAATGCAAAAAGATTTTCAATGACTTGAAAATTGAACAGCTA<br>TACCACGAATATGAAGAGTCTATTGCCAAGGATTTGAAGGCCAAAATTTCTC<br>AGGTCGATGAGTCTCGTGGCTTCAAAGCTGATGTCTTAACTGCGTTCTTGAA<br>CAAAGTTTACAAGAGAAGCAAATAG
P_{TDH3}	TTTGTTTGTTTATGTGTGTTTATTCGAAACTAAGTTCTTGGTGTTTTAAAACTA<br>AAAAAAAGACTAACTATAAAAGTAGAATTTAAGAAGTTTAAGAAATAGATT<br>TACAGAATTACAATCAATACCTACCGTCTTTATATACTTATTAGTCAAGTAGG<br>GGAATAATTTCAGGGAACTGGTTTCAACCTTTTTTTTCAGCTTTTTCCAAATC<br>AGAGAGAGCAGAAGGTAATAGAAGGTGTAAGAAAATGAGATAGATACATG<br>CGTGGGTCAATTGCCTTGTGTCATCATTTACTCCAGGCAGGTTGCATCACTCC<br>ATTGAG<br>GTTGTGCCCGTTTTTTGCCTGTTTGTGCCCCTGTTCTCTGTAGTTGCGCTAAGA<br>GAATGGACCTATGAACTGATGGTTGGTGAAGAAAACAATATTTTGGTGCTGG<br>GATTCTTTTTTTTTCTGGATGCCAGCTTAAAAAGCGGGCTCCATTATATTTAGT<br>GGATGCCAGGAATAAACTGTTCACCCAGACACCTACGATGTTATATATTCTGT

	GTAACCCGCCCCCTATTTTGGGCATGTACGGGTTACAGCAGAATTAAAAGGC<br>TAATTTTTTGACTAAATAAAGTTAGGAAAATCACTACTATTAATTATTTACGTA<br>TTCTTTGAAATGGCAGTATTGATAATGATAAACTCGAACTGAAAAAGCGTGT<br>TTTTTATTCAAAATGATTCTAACTCCCTTACGTAATCAAGGAATCTTTTTGCCT<br>TGGCCTCCGCGTCATTAAACTTCTTGTTGTTGACGCTAACATTCAACGCTAGT<br>AT
T_{ADH1}	TCGGCATGCCGGTAGAGGTGTGGTCAATAAGAGCGACCTCATGCTATACCTG<br>AGAAAGCAACCTGACCTACAGGAAAGAGTTACTCAAGAATAAGAATTTTCG<br>TTTTAAAACCTAAGAGTCACTTTAAAATTTGTATACACTTATTTTTTTATAAC<br>T
tHMG1	ATGGcTGCAGACCAATTGGTGAAAACTGAAGTCACCAAGAAGTCTTTTACTG<br>CTCCTGTACAAAAGGCTTCTACACCAGTTTTAACCAATAAAACAGTCATTTC<br>TGGATCGAAAGTCAAAAGTTTATCATCTGCGCAATCGAGCTCATCAGGACCT<br>TCATCATCTAGTGAGGAAGATGATTCCCGCGATATTGAAAGCTTGGATAAGA<br>AAATACGTCCTTTAGAAGAATTAGAAGCATTATTAAGTAGTGGAAATACAAA<br>ACAATTGAAGAACAAAGAGGTCGCTGCCTTGGTTATTCACGGTAAGTTACCT<br>TTGTACGCTTTGGAGAAAAAATTAGGTGATACTACGAGAGCGGTTGCGGTAC<br>GTAGGAAGGCTCTTTCAATTTTGGCAGAAGCTCCTGTATTAGCATCTGATCGT<br>TTACCATATAAAAATTATGACTACGACCGCGTATTTGGCGCTTGTTGTGAAAA<br>TGTTATAGGTTACATGCCTTTGCCCGTTGGTGTTATAGGCCCCTTGGTTATCGA<br>TGGTACATCTTATCATATACCAATGGCAACTACAGAGGGTTGTTTGGTAGCTT<br>CTGCCATGCGTGGCTGTAAGGCAATCAATGCTGGCGGTGGTGCAACAACTG

	TTTTAACTAAGGATGGTATGACAAGAGGCCCAGTAGTCCGTTTCCCAACTTT<br>GAAAAGATCTGGTGCCTGTAAGATATGGTTAGACTCAGAAGAGGGACAAAA<br>CGCAATTAAAAAAGCTTTTAACTCTACATCAAGATTTGCACGTCTGCAACAT<br>ATTCAAACTTGTCTAGCAGGAGATTTACTCTTCATGAGATTTAGAACAACTAC<br>TGGTGACGCAATGGGTATGAATATGATTTCTAAAGGTGTCGAATACTCATTAA<br>AGCAAATGGTAGAAGAGTATGGCTGGGAAGATATGGAGGTTGTCTCCGTTTC<br>TGGTAACTACTGTACCGACAAAAAACCAGCTGCCATCAACTGGATCGAAGG<br>TCGTGGTAAGAGTGTCGTCGCAGAAGCTACTATTCCTGGTGATGTTGTCAGA<br>AAAGTGTTAAAAAGTGATGTTTCCGCATTGGTTGAGTTGAACATTGCTAAGA<br>ATTTGGTTGGATCTGCAATGGCTGGGTCTGTTGGTGGATTTAACGCACATGCA<br>GCTAATTTAGTGACAGCTGTTTTCTTGGCATTAGGACAAGATCCTGCACAAA<br>ATGTTGAAAGTTCCAACTGTATAACATTGATGAAAGAAGTGGACGGTGATTT<br>GAGAATTTCCGTATCCATGCCATCCATCGAAGTAGGTACCATCGGTGGTGGT<br>ACTGTTCTAGAACCACAAGGTGCCATGTTGGACTTATTAGGTGTAAGAGGCC<br>CGCATGCTACCGCTCCTGGTACCAACGCACGTCAATTAGCAAGAATAGTTGC<br>CTGTGCCGTCTTGGCAGGTGAATTATCCTTATGTGCTGCCCTAGCAGCCGGCC<br>ATTTGGTTCAAAGTCATATGACCCACAACAGGAAACCTGCTGAACCAACAA<br>AACCTAACAATTTGGACGCCACTGATATAAATCGTTTGAAAGATGGGTCCGT<br>CACCTGCATTAAATCCTAA

P_{PGK1}	TGTTTTATATTTGTTGTAAAAAGTAGATAATTACTTCCTTGATGATCTGTAAAA<br>AAGAGAAAAAGAAAGCATCTAAGAACTTGAAAAACTACGAATTAGAAAAG<br>ACCAAATATGTATTTCTTGCATTGACCAATTTATGCAAGTTTATATATATGTAA<br>ATGTAAGTTTCACGAGGTTCTACTAAACTAAACCACCCCCTTGGTTAGAAGA<br>AAAGAGTGTGTGAGAACAGGCTGTTGTTGTCACACGATTCGGACAATTCTGT<br>TTGAAAGAGAGAGAGTAACAGTACGATCGAACGAACTTTGCTCTGGAGATC<br>ACAGTGGGCATCATAGCATGTGGTACTAAACCCTTTCCCGCCATTCCAGAAC<br>CTTCGATTGCTTGTTACAAAACCTGTGAGCCGTCGCTAGGACCTTGTTGTGTG<br>ACGAAATTGGAAGCTGCAATCAATAGGAAGACAGGAAGTCGAGCGTGTCT<br>GGGTTTTTTCAGTTTTGTTCTTTTTGCAAACAAATCACGAGCGACGGTAATTT<br>CTTTCTCGATAAGAGGCCACGTGCTTTATGAGGGTAACATCAATTCAAGAAG<br>GAGGGAAACACTTCCTTTTTCTGGCCCTGATAATAGTATGAGGGTGAAGCCA<br>AAATAAAGGATTCGCGCCCAAATCGGCATCTTTAAATGCAGGTATGCGATAG<br>TTCCTCACTCTTTCCTTACTCACGAGTAATTCTTGCAAATGCCTATTGTGCAG<br>ATGTTATAATATCTGTGCGT
P_{TEF2}	TATACTTACATATAGTAGATGTCAAGCGTAGGCGCTTCCCCTGCCGGCTGTGA<br>GGGCGCCATAACCAAGGTATCTATAGACCGCCAATCAGCAAACTACCTCCGT<br>ACATTCATGTTGCACCCACACATTTATACACCCAGACCGCGACAAATTACCC<br>ATAAGGTTGTTTGTGACGGCGTCGTACAAGAGAACGTGGGAACTTTTTAGGC<br>TCACCAAAAAAGAAAGAAAAAATACGAGTTGCTGACAGAAGCCTCAAGAA<br>AAAAAAAATTCTTCTTCGACTATGCTGGAGGCAGAGATGATCGAGCCGGTA<br>GTTAACTATATATAGCTAAATTGGTTCCATCACCTTCTTTTCTGGTGTCGCTCC

	TTCTAGTGCTATTTCTGGCTTTTCCTATTTTTTTTTTTTCCATTTTTCTTTCTCTCTT<br>TCTAATATATATAAATTCTCTTGCATTTTCTATTTTTCTCTCTATCTATTCTACTTGT<br>TTATTCCCTTCAAGGTTTTTTTTTAAGGAGTACTTGTTTTTAGAATATACGGTC<br>AACGAACTATAATTAACTAAACACTAGTACC
T_{CYC1}	tcatgtaattagttatgtcacgcttacattcacgccctccccccacatccgctctaaccgaaaaggaaggagttagacaacc<br>tgaagtctaggtccctatttattttttttatagttatgttagtattaagaacgttatttatatttcaaatttttcttttttttctgtacag<br>acgcgtgtacgcatgtaacattatactgaaaaccttgcttgagaaggttttgggacgctcgaaggctttaatttgc
L3	AACGACGGTAGACGCCAACTACGCTGACAGACCGATTTGTTTAAGATTAGA<br>AGATTTTTAGCCGCGCCGCAATCGGAACCAGCAAACTCAATTCTGGGAACA<br>GTTTAAAATACTAGTAATTACGATAGCCGAGAAACGGACTAAGTCCGC
T_{FBA1}	ggtaccGCTATCAAAAACGATAGATCGATTAGGATGACTTTGAAATGACTCCGC<br>AGTGGACTGGCCGTTAATTTCAAGCGTGAGTAAAATAGTGCATGACAAAAG<br>ATGAGCTAGGCTTTTGTAAAAATATCTTACGTTGTAAAATTTTAGAAATCATT<br>ATTTCCTTCATATCATTTTGTCATTGACCTTCAGAAGAAAAGAGCCGACCAAT<br>AATATAAATAAATAAATAAAAATAATATTCCATTATTTCTAAACAGATTCAAT<br>ACTCATTAAAAAACTATATCAATTAATTTGAATTAACaagctt
T_{PDC1}	gaattcGCGATTTAATCTCTAATTATTAGTTAAAGTTTTATAAGCATTTTTATGTAA<br>CGAAAAATAAATTGGTTCATATTATTACTGCACTGTCACTTACCATGGAAAG<br>ACCAGACAAGAAGTTGCCGACAGTCTGTTGAATTGGCCTGGTTAGGCTTAA<br>GTCTGGGTCCGCTTCTTTACAAATTTGGAGAATTTCTCTTAAACGATATGTAT<br>ATTCTTTTCGTTGGAAAAGATGTCTTCCAAAAAAAAAAACCGATGAATTAGTG<br>GAACCAAGGAAAAAAAAAGAGGTATCCTTGATTAAGGAACAgagctc

L4	CCAGACGATACAGAGGCTAAGAATAACGCAGATAATCGCTCTAACGAAACG<br>TACTAAAAGATTTCTTTTGAAGTAACTAGATACCCTGGTCTTATACTAGGTAT<br>CTTTGTCAGAAACGGCCTAAGACTACAGTAAGAGCAGTTGGAACCT
